# Characteristics and *in vitro* response of thin hydroxyapatite–titania films produced by plasma electrolytic oxidation of Ti alloys in electrolytes with particle additions

**DOI:** 10.1039/c5ra22178a

**Published:** 2016-02-01

**Authors:** W. K. Yeung, I. V. Sukhorukova, D. V. Shtansky, E. A. Levashov, I. Y. Zhitnyak, N. A. Gloushankova, P. V. Kiryukhantsev-Korneev, M. I. Petrzhik, A. Matthews, A. Yerokhin

**Affiliations:** a University of Sheffield, Mappin Street, Sheffield, S1 3JD, UK. Email: A.Yerokhin@Sheffield.ac.uk; Fax: +44 (0)1142 225943; Tel: +44 (0)1142 225970; b National University of Science and Technology ‘MISiS’, Leninsky prospect 4, Moscow 119049, Russia; c N.N. Blokhin Russian Cancer Research Centre, Kashirskoe shosse 24, Moscow 115478, Russia; d University of Manchester, Oxford Road, Manchester, M13 9PL, UK

## Abstract

The enhancement of the biological properties of Ti by surface doping with hydroxyapatite (HA) is of great significance, especially for orthodontic applications. This study addressed the effects of HA particle size in the electrolyte suspension on the characteristics and biological properties of thin titania-based coatings produced on Ti–6Al–4V alloy by plasma electrolytic oxidation (PEO). Detailed morphological investigation of the coatings formed by a single-stage PEO process with two-step control of the electrical parameters was performed using the Minkowski functionals approach. The surface chemistry was studied by glow discharge optical emission spectroscopy and Fourier transform infrared spectroscopy, whereas mechanical properties were evaluated using scratch tests. The biological assessment included *in vitro* evaluation of the coating bioactivity in simulated body fluid (SBF) as well as studies of spreading, proliferation and osteoblastic differentiation of MC3T3-E1 cells. The results demonstrated that both HA micro- and nanoparticles were successfully incorporated in the coatings but had different effects on their surface morphology and elemental distributions. The micro-particles formed an irregular surface morphology featuring interpenetrated networks of fine pores and coating material, whereas the nanoparticles penetrated deeper into the coating matrix which retained major morphological features of the porous TiO_2_ coating. All coatings suffered cohesive failure in scratch tests, but no adhesive failure was observed; moreover doping with HA increased the coating scratch resistance. *In vitro* tests in SBF revealed enhanced bioactivity of both HA-doped PEO coatings; furthermore, the cell proliferation/morphometric tests showed their good biocompatibility. Fluorescence microscopy revealed a well-organised actin cytoskeleton and focal adhesions in MC3T3-E1 cells cultivated on these substrates. The cell alkaline phosphatase activity in the presence of ascorbic acid and β-glycerophosphate was significantly increased, especially in HA nanoparticle-doped coatings.

## Abstract 2

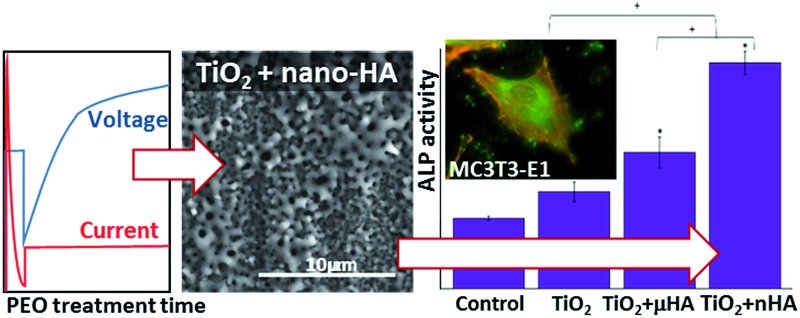
Enhanced incorporation of hydroxyapatite nanoparticles in porous titania coating formed by plasma electrolytic oxidation significantly increases surface osteogenic activity.

## 1. Introduction

Bone consist of an extracellular matrix and the mineral hydroxyapatite (HA) phase. The latter has been well-characterised and widely used in orthodontics to stimulate osteogenesis. However, due to the brittle nature of HA ceramics, there has been growing interest in HA-coated metallic dental implants. In particular, a large number of studies indicated that incorporation of HA into porous coatings improves the biological performance of implants.^1–3^ Plasma electrolytic oxidation (PEO) has been extensively used to form porous coatings on titanium alloys to enhance their bioactivity.^4–8^ The method involves electrolytic treatment at voltages above the dielectric breakdown voltage of the anodic oxide film formed on the metal surface.^9^ However, synthesising crystalline HA with a uniform porous morphology using PEO treatments in calcium-based electrolytes is challenging.^10–13^ Therefore, various post-treatments have been proposed to crystallise or deposit the crystalline HA onto the coatings, including electrodeposition,^14,15^ hydrothermal^16^ and chemical^17^ treatments, which complicates the coating procedure.

Nie *et al.* ^18^ suggested using sequential treatments with PEO followed by electrophoretic deposition of HA. However, this alters the porous morphology of PEO coatings and can potentially increase the risk of the top HA layer wearing off or delaminating during the implant insertion. Moreover, the use of non-biocompatible organic compounds to stabilise the electrolyte suspension remains a concern. Kazek-Kęsik *et al.* ^19^ used various electrolyte suspensions to form bioactive titania coatings doped with Ca_3_(PO_4_)_2_, CaSiO_3_, and SiO_2_ by a single-stage PEO process. Previous work involving some of the authors^20^ has demonstrated that by using a single-stage PEO process with a two-step control of electrical parameters it was possible to *in situ* incorporate HA micro-particles from the electrolyte suspension into the growing coating, without the need for stabilisers, and this appeared to influence the coating morphology and dielectric behaviour. It was suggested^20^ that incorporation of HA nanopowder with the two-step control method could form a bi-layer surface structure with an inner dense barrier layer and an outer porous layer, the morphology of the latter being similar to that of bioactive TICER® and TiUnite® surface finishes.^6,21–23^ Furthermore, incorporation of HA nanopowder has been proposed to be more beneficial for osteoblastic proliferation when compared to HA micro-powder^24^ as it is more readily absorbed into cells.^25^ However, with the previous studies^20^ having been focused primarily on electrochemical aspects of electrolytic plasma processing and resulting coating behaviour, the crucial question remains regarding the effects of HA particle size in the two-step control PEO process on the coating characteristics and biological properties. Therefore, this study focuses on comparing the surface morphology, mechanical properties and osteoblastic response of PEO coatings formed by the two-step control method in electrolytes with HA micro- and nanoparticle additions.

## 2. Experimental methods

### 2.1. Sample preparation

Titanium alloy Ti–6Al–4V disks, 15 mm in diameter and 5 mm thick, were polished using abrasive paper with 240 to 1200 grit to achieve a surface finish with arithmetic average roughness of *R*_a_ ≈ 0.1 μm. The samples were subsequently cleaned in acetone, isopropanol and distilled water, then air-dried. The PEO treatments were carried out in a 6 g l^–1^ disodium hydrogen phosphate (99% Na_2_HPO_4_, Fisher Scientific) electrolyte (pH = 9.2; *κ* = 6.3 mS cm^–1^) with 20 g l^–1^ additions of HA micro-powder (the mean average particle size of 2.5 μm, Fluidinova) or 10 g l^–1^ addition of HA nanopowder (nanoXIM-Hap303, Moreira da Maia, Portugal). The powders were slowly added in the electrolyte under vigorous stirring to avoid agglomeration. Then the solutions were stirred for at least an hour to allow particle hydration and stabilisation of electrolyte properties.

The PEO treatment was performed using the two-step control method, wherein a potentiostatic anodic polarisation, *U* ^+^ = 250 V was applied to the surface for 15 to 30 s to allow surface passivation to occur, then galvanostatic control at a mean average anodic current density of *i* ^+^ = 300 mA cm^–2^ was employed using pulsed unipolar (PUP) or pulsed bipolar (PBP) mode. Table 1 shows processing parameters employed for individual treatments, with justification provided elsewhere.^20^

**Table 1:** Electrical conditions of PEO treatments

Sample	Step 1	Step 2^*a*^				
	*U* ^+^ (V)	*i* ^–^/*i* ^+^	*τ*_ON_^+^	*τ*_OFF_^+^	*τ*_ON_^–^	*τ*_OFF_^–^
TiO_2_	250	0	400	100	—	—
TiO_2_ + *μ*HA		1/3	400	25	50	25
TiO_2_ + *n*HA		1/2	400	25	50	25

^*a*^ *τ*_ON_ and *τ*_OFF_ refer to the durations (μs) of corresponding pulse and pause.

### 2.2. Surface characterisation

A JEOL JSM-6400 scanning electron microscope (SEM) with an energy dispersive X-ray (EDX) spectroscopy attachment was employed to analyse the surface morphologies and elemental composition of the coatings. Prior to structural characterisation, the samples were cleaned and carbon coated to minimise surface charging during imaging. The thickness measurements (*n* = 10) were conducted with an Elcometer 355 modular thickness gauge system equipped with a standard no. 4 Anodisers probe. Surface roughness (*R*_a_) was measured using a Veeco Dektak 1500 surface profilometer. The scans were performed in a standard ‘Hills and Valleys’ mode with stylus force of 3 mg and scan length of 4000 μm (*n* = 3).

Fourier Transform Infrared (FTIR) spectroscopy analysis was carried out using a Vertex 70v vacuum spectrometer (Bruker) operated in an attenuated total reflectance (ATR) mode, in the range of 400–3000 cm^–1^ with a resolution of 4 cm^–1^. The elemental depth profiles were determined using a Profiler 2 glow discharge optical emission spectrometer (GDOES, Horiba Jobin Yvon) equipped with a *∅* 4 mm anode, operating with a radio frequency discharge at a pressure of 700 Pa and a power of 35 W.

X-Ray diffraction (XRD) patterns were recorded using a D8 Advance X-ray diffractometer (Bruker) utilising CuKα monochromatic radiation. The scans have been performed in 2*θ* range of 10° to 90° in both conventional Bragg–Brentano and Seeman–Bohlin (GIXRD) geometry with grazing incidence of 3°.

### 2.3. Calculations of Minkowski measures

The Minkowski functionals were used to derive and compare quantities describing morphological features of different PEO coatings.^26^ The binary SEM images were characterised in terms of surface coverage (*C*), the boundary length (*L*) and the Euler number (*E*), corresponding to the amount of pores (dark) and surface (bright) areas, the length of boundaries between them and the connectivity of the pores, respectively. As these functionals vary between images, the results were normalised using methods proposed by Toccafondi *et al.* ^26^ and then plotted as functions of binarisation threshold values (*ρ*). These quantities were calculated in MATLAB with source code developed by Salerno and Banzato.^27^

### 2.4. Scratch test

Coating adhesion was evaluated using a REVETEST scratch tester (CSM Instruments). The normal load (*L*) on a diamond Rockewell C indenter with a tip curvature radius of about 200 microns was progressively increased from 0.9 N to maximum load of 30 N. This corresponds to a loading rate of 29.1 N min^–1^. The sample was moved at a rate of 5 mm min^–1^, the scratch length was about 5 mm. After the test, the scratch on the coating surface was examined using an optical microscope (×800) to determine the characteristic events of failure. Acoustic emission (AE) and friction coefficient (FC) were also registered during the tests and analysed subsequently to help determining critical loads causing the failure.

### 2.5. Biocompatibility

#### 2.5.1. *In vitro* bioactivity study in simulated body fluid (SBF)

The coated samples were immersed in flasks filled with 40 ml of SBF (Table 2) and soaked at 36.7 °C for 21 days, with the solution being replaced every seven days. The samples were removed from SBF on days 7, 14, and 21, sonicated and studied using SEM, EDX and XRD methods.

**Table 2:** Composition of simulated body fluid

Reagent	Amount^*a*^ (g l^–1^)
Sodium chloride (NaCl)	7.996
Sodium bicarbonate (NaHCO_3_)	0.350
Potassium chloride (KCl)	0.224
Potassium phosphate dibasic trihydrate (K_2_HPO_4_·3H_2_O)	0.228
Magnesium chloride hexahydrate (MgCl_2_·6H_2_O)	0.305
1 M hydrochloric acid (HCl)	40 ml
Calcium chloride (CaCl_2_)	0.278 g
Sodium sulfate (Na_2_SO_4_)	0.071
Tris(hydroxymethyl) aminomethane (C_4_H_11_NO_3_)	6.057

^*a*^Unless otherwise indicated.

#### 2.5.2. Cell culture

Mouse osteoblastic cells (MC3T3-E1, ATCC) were used. The samples were sterilised in 70% ethanol, washed in phosphate buffered saline (PBS) to remove residue substances and placed in 24-well culture plates. The cells were seeded with a density of 6 × 10^4^ cells ml^–1^ into plates with DMEM/F12 medium (Invitrogen) supplemented by 10% fetal bovine serum (PAA Laboratories) and incubated at 37 °C in a humidified incubator containing 5% of CO_2_. Coverslips were used for comparison.

#### 2.5.3. Actin cytoskeleton focal adhesion staining and morphometric analysis

In order to visualise the actin cytoskeleton and focal adhesions, immunostaining was used. Cells incubated for 24 h were washed in PBS and fixed with 3.7% formaldehyde for 10 min and washed with PBS again. The fixed cells were permeabilised in 0.5% Triton-X100 and immersed in primary antibodies for paxillin (BD Transduction Lab) for 40 min followed by incubation with TRITC-phalloidin and Alexa488-conjugated goat anti-mouse IgG secondary antibodies (Molecular Probes). The samples were examined using an Axioplan microscope (ZEISS) equipped with a Plan-Neofluar 40 × 0.75 objective and Olympus DP-100 camera. ImageJ software was used to determine the average area of individual cells cultivated on the samples, 30 cells in total were analysed per sample.

#### 2.5.4. Proliferation assay

The standard quantity of osteoblasts (7 × 10^3^ cell per cm^–2^) was seeded on the coated and control samples which were then placed into 24-well culture plates and cultivated in DMEM/F12 medium with 10% FCS. After one, three, five, and seven days, cells were fixed by 3.7% formaldehyde, permeated with Triton X-100 and stained with DAPI (Sigma, USA). The number of cells in the field was evaluated using an Axioplan microscope (Zeiss) equipped with 40× objective. The cell numbers reported here represent the mean average values derived from measurements in 30 different areas on each sample.

#### 2.5.5. Alkaline phosphatase (ALP) assay

The samples were placed into 24-well culture plates and cells were seeded with a density of 8 × 10^4^ cells per ml in α-MEM supplemented with 10% fetal bovine serum (PAA Laboratories). The next day, the culture medium was replaced with α-MEM supplemented with 10% fetal bovine serum, 50 μg ml^–1^ ascorbic acid, and 10 mM β-glycerophosphate (Sigma), and incubated at 37 °C in a humidified incubator containing 5% of CO_2_. The medium was changed every three days. After culturing for 14 days, the samples were washed with Earle's Balanced Salt Solution and transferred into another plate. The cells were detached from samples with a trypsin/EDTA solution, washed with PBS three times, then re-suspended in 200 μl of 0.1% Triton X-100, 10 mM Tris-Cl (pH = 7.5), 1 mM MgCl_2_ and underwent 4 cycles of freeze–thaw and sonication. After that, 100 μl of *p*-nitrophenyl phosphate (pNPP) was added to 10 μl of cell lysate followed by incubation for 30 min at 37 °C. The reaction was stopped by adding 0.4 M of NaOH and the plate was read at a wavelength of 405 nm by Stat Fax 3200 microplate reader (Awareness Technology Inc). The ALP activity was determined as a rate of *p*-nitrophenol liberation from *p*-nitrophenyl phosphate and expressed as nmol of *p*-nitrophenol formation per minute per milligram of cellular protein. All measurements were reported as the mean average value ± standard deviation.

#### 2.5.6. Statistical analysis

Statistical analysis of data was performed using two-way ANOVA to test differences between samples. Differences were considered as significant when the *p*-value was less than 0.05.

## 3. Results and discussion

### 3.1. Characteristics of PEO process

**Fig. 1:**
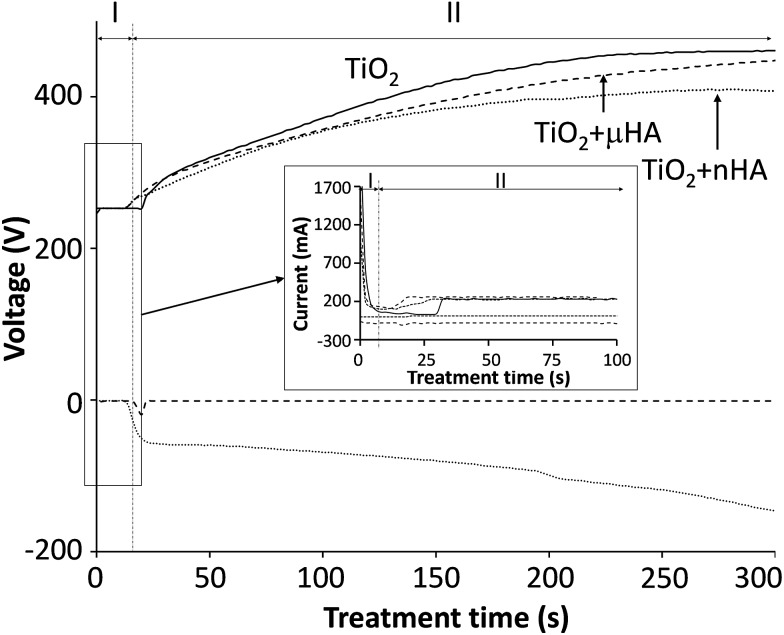
Electrical transients recorded during PEO treatments of Ti–6Al–4V alloy in 6 g l^–1^ aqueous solution of Na_2_HPO_4_ alone (TiO_2_) and with addition of either 20 g l^–1^ of *μ*HA (TiO_2_ + *μ*HA) or 10 g l^–1^ of *n*HA (TiO_2_ + *n*HA).

Fig. 1 reveals the voltage transients recorded during the two-step control PEO treatment. The transients are categorised into two stages, where stage I corresponds to the potentiostatic mode, with 250 V applied for 15 to 30 s and stage II was carried out under the galvanostatic control. This is based on the results from previous work,^20^ indicating that the two-step control method allows more stable voltage growth into the region where sparking commences. Interestingly, when comparing voltage transients for the treatments in electrolytes with *μ*HA and *n*HA additions, it can be seen that the size of HA powder in the electrolyte has an influence to the voltage growth rate, resulting in it being stabilised comparatively earlier when *n*HA was used, with the final voltage reaching only 408 V (*versus* 461 V in the case of *μ*HA). This tendency persisted in both PUP and PBP PEO treatments, indicating that electrolyte and/or surface properties, rather than differences in the electrical regime employed, should be primarily responsible for such behaviour. Since neither of the HA additions affected the electrolyte pH and conductivity, it is reasonable to assume the voltage behaviour reflected changes in the coating characteristics. Gao *et al.* ^28^ observed strong correlation between final voltage and thickness of PEO coatings on Mg. In our case however, the coating thickness showed no statistically significant difference between the batches, always being in the range of 7.5 to 9.5 μm. This indicates that the observed voltage behaviour could be due to incorporation of HA phases with different dielectric properties^29,30^ into TiO_2_ matrix of the growing PEO coating. Moreover, a more abundant incorporation of *n*HA should be expected, even though its concentration in the electrolyte has been lower than that of *μ*HA.

### 3.2. Surface morphology

**Fig. 2:**
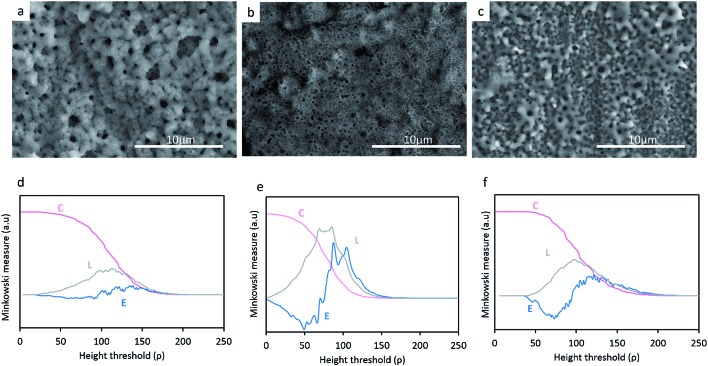
SEM micrographs of (a) TiO_2_, (b) TiO_2_ + *μ*HA and (c) TiO_2_ + *n*HA and the respective Minkowski functionals (d–f) *C*(*ρ*), *L*(*ρ*) and *E*(*ρ*).

Fig. 2a–c reveals the surface morphology of the TiO_2_, TiO_2_ + *μ*HA and TiO_2_ + *n*HA coatings, with corresponding Minkowski functional plots shown in Fig. 2d–f. It can be seen that the surface morphology remains uniformly porous with incorporation of HA particles. Meanwhile, pores appear to be filled by deposits on the TiO_2_ + *μ*HA coating, most probably HA micro-particles. The addition of HA powder with different sizes appears to influence the average pore size in the coating. In particular, the pore size of the TiO_2_ + *μ*HA coating is lower than that of the TiO_2_ + *n*HA coating.

When compared the Minkowski plots (Fig. 2d–f), the shape of surface coverage *C* curves is not significantly different for the studied coatings, indicating they all have similar porous features. However, the curve inflection occurs within different threshold ranges, *ρ* = (60–130), (40–120) and (60–160) for the TiO_2_, TiO_2_ + *μ*HA and TiO_2_ + *n*HA coatings respectively. This implies that the coating containing HA microparticles possess a relatively higher porosity, whereas the one with nanoparticles may have some pores partly filled, which is consistent with corresponding SEM images.

The boundary length *L* curves of both TiO_2_ and TiO_2_ + *n*HA coatings show symmetrical maxima, suggesting a presence of certain regularities in the pore size and shape distribution for these coatings.^26^ A higher *L*_max_ value for the latter coating implies a more developed interconnectivity between the pores and the matrix. At the same time, the *L* curve of the TiO_2_ + *μ*HA coating features significant distortions, which indicates the surface morphology is highly irregular, with pores possibly deviating from circular shape and interacting with surface roughness to form interpenetrating networks between the voids and the coating material. This is consistent with corresponding Euler curve (*E*) which indicates high connectivity of the pore space. Several positive maxima can be observed in the *E* curve of the TiO_2_ coating, which suggests a presence of isolated regions of punctiform structure.^31^ In contrast, the two-lobbed shape of the *E* curve for the TiO_2_ + *n*HA coating resembles that of a structure with regular ordered porous array.^26^

**Fig. 3:**
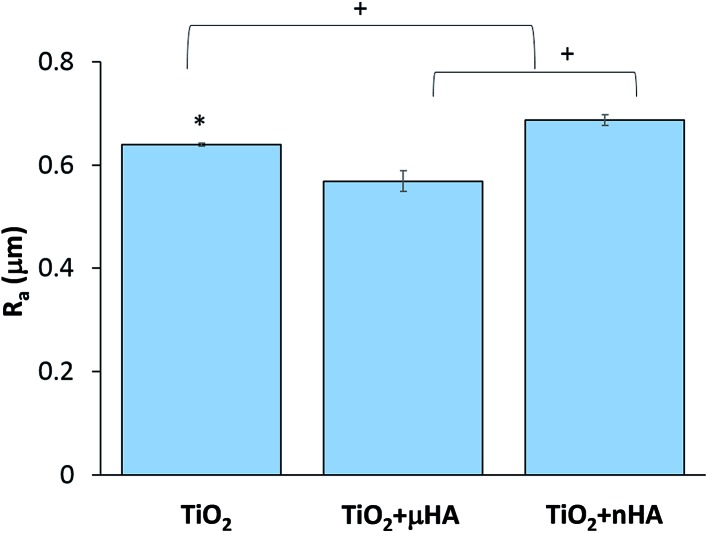
The mean average values of surface roughness *R*_a_ of TiO_2_, TiO_2_ + *μ*HA and TiO_2_ + *n*HA coatings. Symbols * and + mean significant differences with TiO_2_ + *μ*HA and TiO_2_ + *n*HA respectively (*p* < 0.05).

Fig. 3 reveals the average roughness values for TiO_2_, TiO_2_ + *μ*HA and TiO_2_ + *n*HA coatings. Results show the incorporation of *n*HA significantly increases the surface roughness when compared to those of TiO_2_ and TiO_2_ + *μ*HA coatings. This complies with Fig. 2, where the *C* curve for the TiO_2_ + *n*HA coating (Fig. 2f) shows a more distinctive sigmoidal shape. At the same time, introduction of *μ*HA reduces *R*_a_, probably due to blockage of pore openings by relatively large hydroxyapatite particles, as signified by Fig. 2b. Despite statistical significance of difference between the average values of surface roughness, the overall roughness variation range is rather narrow, 0.57 to 0.69 μm.

### 3.3. Chemical composition

#### 3.3.1. Elemental distribution across the coating thickness

**Fig. 4:**
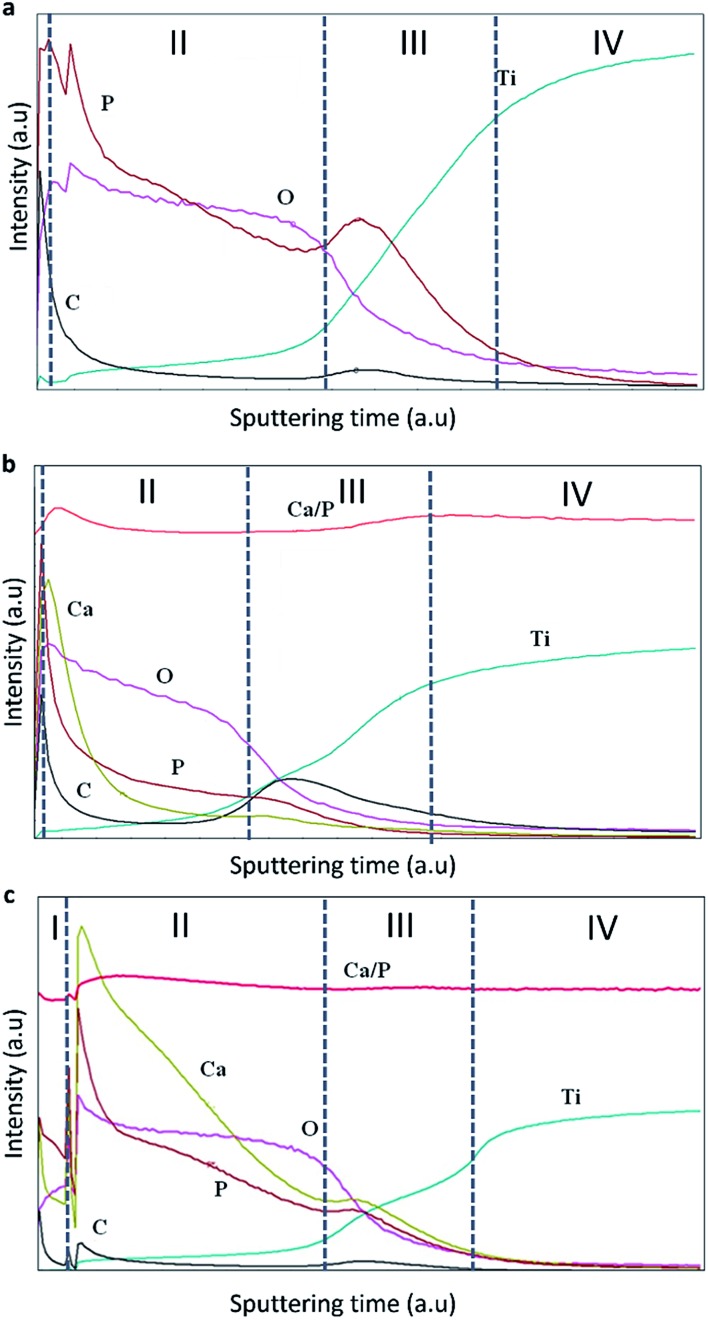
Elemental distributions in the studied coatings (a) TiO_2_, (b) TiO_2_ + *μ*HA, and (c) TiO_2_ + *n*HA.

Fig. 4 reveals elemental distribution profiles over the thickness of the TiO_2_, TiO_2_ + *μ*HA and TiO_2_ + *n*HA coatings. Disturbances in the elemental curves at the initial stage of sputtering, especially for the TiO_2_ + *n*HA coating (Fig. 4c), are associated with surface roughness and air penetration into the porous PEO structure.^32^ This agrees well with results of morphological analysis (Fig. 2) and surface roughness evaluation (Fig. 3) where TiO_2_ and TiO_2_ + *n*HA coatings were shown to have the highest surface roughness. As the analysis progresses, the surface is etched and the roughness is decreased, so the disturbance is reduced.

Four zones can be identified in the elemental profiles. The first zone, corresponding to the beginning of the analysis, is characterised by an elevated carbon and phosphorus content. This was probably due to the presence of dissolved CO_2_ in the aerated alkaline electrolyte, which would result in adsorption of carbonate anions in the porous surface structure during the PEO treatment, with subsequent release of carbon in the initial stage of GDOES analysis. Similarly, the increased content of P on the coating surface of TiO_2_ coating can be explained by adsorption of phosphate anions. When compared the elemental profiles of the TiO_2_ + *μ*HA and TiO_2_ + *n*HA coatings, an elevated calcium level can also be observed, confirming the incorporation of HA within the coating. The elevated P content at the outer region of the TiO_2_ + *μ*HA coating could thus be a combination of phosphate adsorption and HA incorporation.^33^

The second zone is characterised by gradual reduction of P, O (and Ca, in the case of HA-containing coatings) and corresponding increase in Ti content. This is likely to be attributed to phosphate and HA deposition on wavy porous surfaces of PEO coatings, creating ‘diffusion tail’ effects in elemental profiles during GDOES analysis. Other possible contributing factors could include effects associated with non-ideal shape of crater and concentration gradients present in the coatings, but these are less likely to be significant at such length scales. As regards to the HA-containing coatings, Fig. 4b shows Ca is predominantly distributed in the outer coating region, suggesting the size of HA micro-particles is too large to penetrate in the pores, so the particles are accumulated at the surface. This agrees with Fig. 2b, where the surface of TiO_2_ + *μ*HA coating appears to represent a superposition of original rough TiO_2_ coating with fine porous deposit. However this is different from a distribution of similar size SiO_2_ microparticles, clustering throughout the PEO coating produced on AM50 Mg alloy under potentiostatic polarisation at 450 V by Lu *et al.* ^34^ This is likely to be due to much higher current densities developed under such conditions at the earlier stages of the coating growth; this would be undesirable in our case as it could cause dehydration or thermolysis of HA particles.

When *n*HA has been used, Ca was detected throughout the coating, along with high P content (Fig. 4c). This suggests enhanced incorporation of HA nanoparticles in the PEO coating, and the nanoscale size of particles suspended in the electrolyte is preferable to penetrate through the porous structure, which agrees well with both characteristics of the PEO process discussed in Section 3.1 and the coating morphology observed in Fig. 2c. Moreover, this outlines the main advantage of using nanoparticle suspensions as electrolytes for PEO process, which allows decoupling HA incorporation from formation of the porous structure in the coating matrix. Direct HA synthesis from electrolytes containing soluble Ca and P salts usually requires solutions with over-stoichiometric Ca/P ratio (≫1.67)^11,12,35^ that may be unstable due to the tendency for spontaneous HA nucleation or harsh polarisation conditions^11,12,36,37^ that could adversely affect structure and phase composition of the formed deposits. In contrast, the nanoparticle suspensions are stable and allow operation at softer electrical regimes, which mitigates the risk of HA decomposition in the plasma discharge during the coating growth.

The third zone is characterised by enrichment in P content, which is associated with the inner barrier layer of the coating. This is accepted to be due to phosphate anions attracted to the substrate by the positive bias, being able to penetrate *via* open porosity all the way through to the interfacial barrier region. It can also be observed that C content increased noticeably in the inner region associated with the barrier layer, indicating similarities in behaviour of carbonate and phosphate anions during PEO process. For the TiO_2_ + *n*HA coating, it must be noted that both Ca and P contents in the third zone increase in a proportional manner (Fig. 4c). This suggests the presence of *n*HA at the barrier layer of that coating, which is not observed in the TiO_2_ + *μ*HA coating. This can be explained as follows. Since the isoelectric point of hydroxyapatite is about 7.3,^38^ HA particles suspended in an alkaline solution would attain negative charge and, during PEO processing, migrate towards positively biased metal substrate. Both charge and mobility of hydrated HA particles would increase with decreasing their size, resulting in HA nanoparticles being able to migrate through the porous coating more readily compared to the micro-particles. Finally, the fourth zone refers to the gradual transition to the titanium substrate.

#### 3.3.2. Surface chemical bonding

**Fig. 5:**
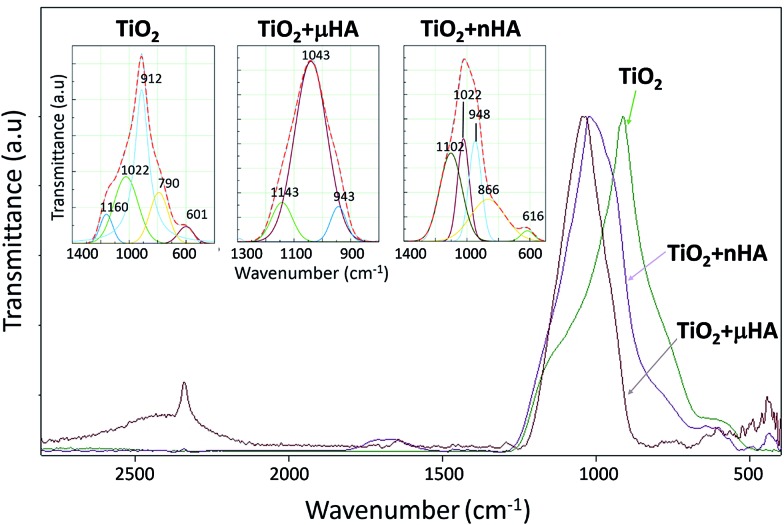
ATR-FTIR spectra of TiO_2_, TiO_2_ + *μ*HA, and TiO_2_ + *n*HA coatings.

ATR-FTIR spectra of the studied coatings are shown in Fig. 5. The characteristic bands of phosphate groups were reported to appear at 460, 560–600, 960, and 1020–1120 cm^–1^.^39^ A broad band can be observed in the 1400–400 cm^–1^ range due to the presence of different PO_4_ tetrahedra.^40^ The *μ*HA- and *n*HA-doped surfaces demonstrate a strong characteristic band centred at 1050–1200 cm^–1^ and small features at 445 and 560–600 cm^–1^ corresponding to the (PO_4_)^3–^ vibrations. This confirms incorporation of HA micro- and nanoparticles in the TiO_2_ matrix during PEO process. A small sharp peak centered at 2345 cm^–1^ can be distinguished in the TiO_2_ + *n*HA coating, which is probably a result of CO_2_ adsorption.^41^ In the case of TiO_2_, a broad asymmetrical peak was observed in the range of 690–1200 cm^–1^ with a maximum at 900 cm^–1^. Assuming that pure anatase has a one peak at 694 cm^–1^ and rutile has two peaks at 656 and 528 cm^–1^, the contribution of the TiO_2_ phase in the FTIR spectrum appears to be small. This is in a good agreement with the results of Khan *et al.* ^42^ obtained for PEO titania coatings formed in Na_3_PO_4_ solutions. The IR spectrum from the TiO_2_ sample was deconvoluted into 5 peaks centered at 601, 790, 912, 1022, and 1160 cm^–1^ (Table 3). These frequencies can be assigned to the asymmetric and symmetric stretching modes of the P–O–P and O–P–O linkages.^43–45^ The most remarkable feature of the TiO_2_ + *n*HA coating is a presence of well-resolved low intensity bands at 463, 493, and 527, and 560 cm^–1^ (Fig. 5). Literature data available for phosphate glasses indicate a likelihood of the following vibrations of phosphate species manifested in this frequency range (cm^–1^): 462 (O=P–O),^46^ 480 (O–P–O),^43^ 500 (O=P–O),^43,46^ and 530 cm^–1^ (PO_4_^3–^).^44,47^

**Table 3:** Peak position derived from deconvolution of peaks at 500–1300 cm^–1^ in Fig. 5

Peak ID	Coating ID		
	TiO_2_	TiO_2_ + *μ*HA	TiO_2_ + *n*HA
PO_4_^3–^	601	—	616
P–O–P	790	—	866
PO_4_^3–^	912	943	948
PO_4_^3–^	1022	1043	1022
O–P–O	1160	1143	1102

### 3.4. Mechanical properties

**Fig. 6:**
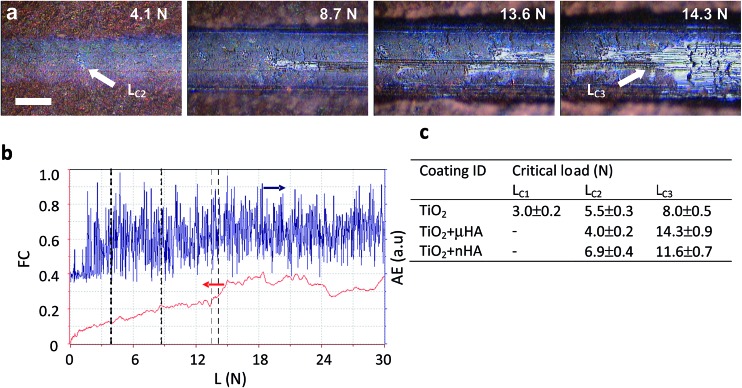
Summary of scratch tests results: (a) typical appearances of the scratch track at characteristics loads in the case of TiO_2_ + *μ*HA coating; (b) corresponding dependencies of friction coefficient and acoustic emission *versus* normal load; (c) derived values of critical loads for the studied coatings. The scale bar in (a) corresponds to 50 μm.

Scratch tests are useful for evaluation of abrasion resistance, cohesive and adhesive strength of PEO coatings on Ti.^4,48^ These characteristics are particularly important for the coatings on dental implants that are inserted by self-tapping and experience high interfacial shear stress due to the different from bone elastic properties. Fig. 6 provides a summary of scratch test results, including examples of scratch morphology for the TiO_2_ + *μ*HA coating (Fig. 6a) and corresponding AE and FC data (Fig. 6b). The former is more sensitive to the instances of cohesive failure within the coating, whereas the latter – to the coating perforation events.^49^ Optical analysis revealed formation of first fine cracks inside the scratch of the TiO_2_ coating at 3 N, which is identified as critical load *L*_C1_ in Fig. 6c. This is in reasonably good agreement with the results of de Souza *et al.*,^48^ who observed cohesive failure of thin TiO_2_ based PEO coatings on Ti at loads < 1 N during scratch tests using sharp Berkovich indenter. In the case of *μ*HA- (Fig. 6a) and *n*HA-doped TiO_2_ coatings, no cracks were observed at low loads, however, a large number of discrete AE spikes in this region (Fig. 6b) indicated development of cohesive fracture in those coatings too. The absence of visual evidence of cracking in HA-doped coatings can be explained by smearing of softer HA phases over the coating surface thereby masking the cracks at low loads. Since all the coatings exhibit abundant porosity, material micro chipping at the pore edges may be responsible for the high amplitude of AE peaks. At higher applied loads, the coating material is displaced to the periphery of the scratch and larger transverse cracks developed in the tensile zone behind the indenter become evident. When the critical load *L*_C2_ is reached, small areas of Ti alloy substrate appear inside the scratch and upon the achievement of *L*_C3_, the substrate becomes exposed across most of the scratch width, with FC changing its behaviour. Due to the low coating thickness and relatively high roughness, both on the surface and at the interface, the values of *L*_C2_ are largely scattered in the range of 4 to 7 N, whereas the *L*_C3_ values show a more distinct separation between pure TiO_2_ and HA-doped coatings (8 N *versus* 11.5 to 14.5 N). This suggests doping with softer HA phases has a positive effect on the scratch resistance of porous TiO_2_ coatings. No evidence of adhesive failure, such as coating spallation or delamination, indicates cohesive failure was the main fracture mechanism for all studied PEO coatings. This is typical for surface treatments resulting in a transition zone between the modified surface layer and the substrate.^50^ A similar role in preventing adhesive failure of the studied coatings may have been played by a combination of high interfacial roughness with a thin barrier layer tightly bonded to the substrate, which is characteristic of PEO coatings on Ti.^7,20^

### 3.5. *In vitro* biological response

**Fig. 7:**
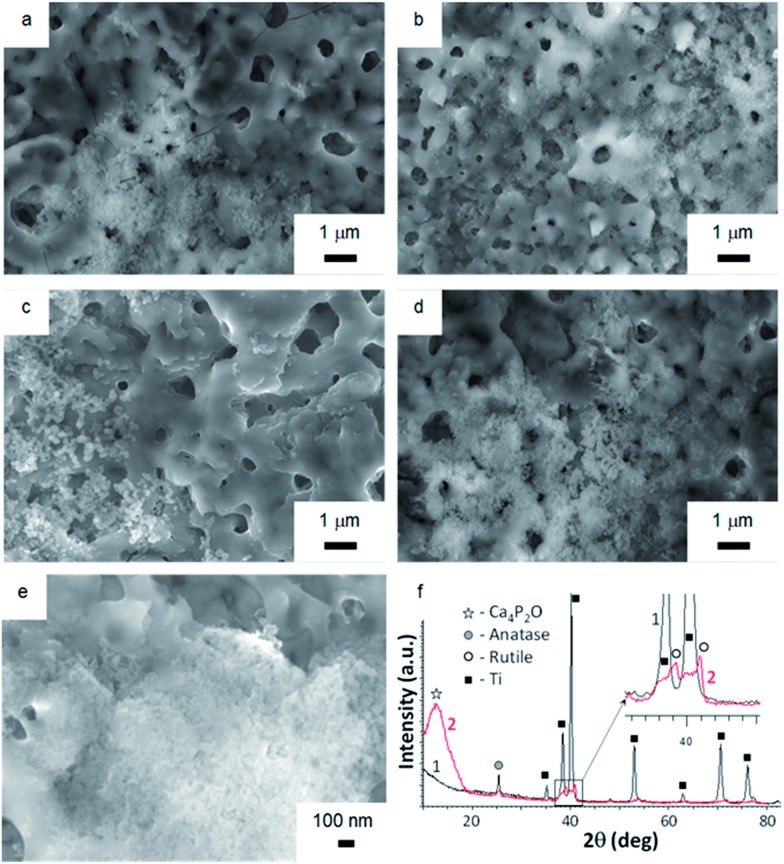
Surface SEM images of (a and c) TiO_2_, (b and d) TiO_2_ + *n*HA, and (e) TiO_2_ + *μ*HA samples after exposure in SBF for (a and b) 7 and (c–e) 21 days. XRD (1) and GIXRD (2) patterns (f) of TiO_2_ + *μ*HA sample after exposure in SBF for 21 days.

**Fig. 8:**
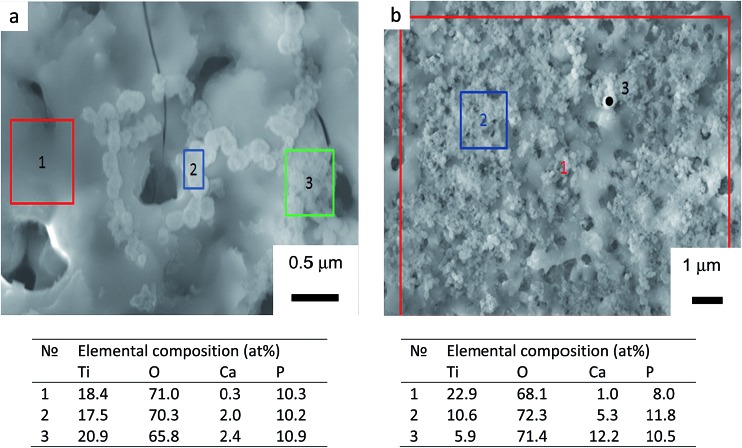
SEM images and EDX analysis of samples with (a) TiO_2_ and (b) TiO_2_ + *n*HA coatings after exposure in SBF for 14 days. Tables under the micrographs show elemental compositions of selected surface regions.

Soaking biomaterials in SBF and studying the kinetics of bone-like apatite layer formation on their surfaces are regarded as the most appropriate approach to estimate their bioactivity *in vitro*.^51,52^ Surface morphologies of TiO_2_, TiO_2_ + *μ*HA, and TiO_2_ + *n*HA coated samples immersed into SBF for 7 and 21 days are presented in Fig. 7. The images of initial sample surfaces are shown in Fig. 2a–c. Exposure of TiO_2_ coating in SBF for 7 days resulted in formation of individual colonies of apatite precipitates with particle size in the range of 0.1 to 0.3 μm (Fig. 7a). Nevertheless, most of the TiO_2_ sample surface was free from such precipitates. In contrast, entire surface of the *n*HA-doped coating was densely populated with fine apatite precipitates, <100 nm in size (Fig. 7b). The results of EDX analysis after 14 days of immersion in SBF demonstrated that the precipitates are Ca-rich phase (Fig. 8). This indicates an initial stage of CaP-phase crystallisation on the surface of PEO coatings. After exposure in SBF for 21 days, the surface of *n*HA-doped coating was completely covered with a relatively thick apatite layer with particle size of 0.1 to 0.3 μm (Fig. 7d). The TiO_2_ + *μ*HA coated sample revealed a dense snow-like apatite layer with particle size < 50 nm (Fig. 7e). In the case of the TiO_2_ coating, numerous particle-free areas were still observed, even after exposure for 21 days, whereas the size and density of apatite particles remained unchanged (Fig. 7c).

Fig. 7f compares the XRD and GIXRD patterns of the TiO_2_ + *μ*HA coating after exposure in SBF for 21 days. Apart from the Ti substrate peaks in the XRD pattern, (101) reflection of TiO_2_ phase (anatase) at 25.5° 2*θ* can be seen. In the GIXRD mode, the substrate peaks completely disappeared while additional peaks appeared in the range of 38° to 42° 2*θ* and were identified as belonging to a TiO_2_ (rutile) phase (Fig. 7f (inset)). Note that titania films, regardless of the fraction of anatase and rutile, were reported to be bioactive due to the presence of surface Ti–OH groups.^53^ The most remarkable feature of the GIXRD pattern is that a broad high intensity peak can be seen in the range of 2*θ* from 10° to 15°. The peak position is consistent with location of the peak corresponding to (002) planes of tetragonal calcium phosphate phase (Ca_4_P_2_O, ICDD cards ## 88-1320, 80-0410, and 44-0368). The obtained results agree well with those previously reported for multicomponent TiCaPCON-based bioactive surfaces.^54^

**Fig. 9:**
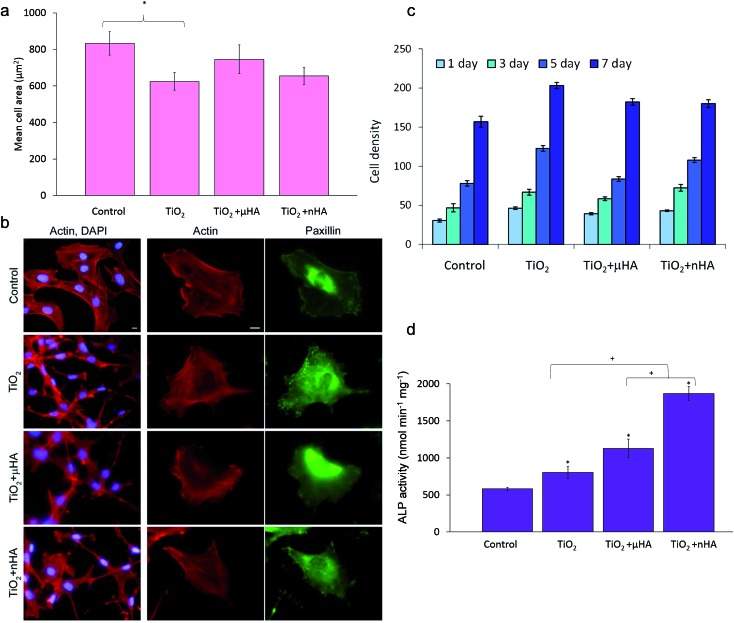
Coatings biocompatibility analysis: (a) morphometric analysis of MC3T3-E1 cells after 24 h in culture on control and PEO coated surfaces. Projected mean cell area from 30 cells on random fields; (b) immunofluorescent staining images of MC3T3-E1 cells for actin (red) and paxillin (green). (c) Proliferation of MC3T2-E1 cells on different surfaces; (d) ALP activity of cells cultured for 14 days. Scale bars in (b) correspond to 10 μm. * means comparison with control and shown *p* < 0.05; + means comparison to TiO_2_ + *n*HA and shown *p* < 0.05.

Results of *in vitro* biological tests involving osteoblastic cells employed in this study are summarised in Fig. 9. Fig. 9a shows mean areas of cells cultivated on the sample surfaces after 24 h. It can be seen that the difference in cell spreading between the TiO_2_, TiO_2_ + *μ*HA, and TiO_2_ + *n*HA coatings is insignificant, whereas the cells on the TiO_2_ coating spread significantly less than those on control.

Fig. 9b illustrates that cells attached to the PEO coated surfaces have well organised actin cytoskeleton and numerous focal adhesions. Cells on both control and PEO coated samples have parallel or radially oriented actin bundles that fill the cytoplasma. This indicates good biocompatibility of all PEO coated surfaces, which is also in good agreement with other work.^36^

Cell proliferation kinetics is a very important aspect of biocompatibility allowing the cytotoxicity of new materials to be determined. Fig. 9c shows comparative proliferation dynamics during the period of 7 days for MC3T3-E1 cells on the TiO_2_, TiO_2_ + *μ*HA, TiO_2_ + *n*HA coatings and the uncoated coverslip after staining with DAPI. The results demonstrate positive dynamics in cell proliferation, indicating absence of cytotoxic effects on all studied PEO coatings.

Alkaline phosphatase provides an early marker of osteoblast differentiation and, therefore, the ALP activity test is a useful method to evaluate the osteoinductive characteristics of materials and their potential ability to stimulate the growth of hard tissue in the process of implant osseointegration. Fig. 9d shows the ALP activity of the MC3T3-E1 cells after culturing for 14 days on the surfaces of the studied coatings. The ALP activity in the cells on all PEO coated samples is significantly higher than that on the control. This indicates that PEO coatings would be generally beneficial for osteoblast differentiation, regardless of differences in their chemistry and surface morphology. This may be related to the nanocrystalline TiO_2_ matrix formed by the PEO treatment of Ti alloy.^7,36,55^ Furthermore, the ALP activity within the PEO coatings increases in the following order: TiO_2_ < TiO_2_ + *μ*HA < TiO_2_ + *n*HA. This indicates that the role of surface chemical composition at this stage increases dramatically. Thus, between the two coatings with similar morphology (TiO_2_ and TiO_2_ + *n*HA), the one doped with *n*HA significantly promotes the ALP activity of cells (*p* < 0.05). At the same time, this effect is less pronounced on the coating doped with HA microparticles. HA has been suggested to enhance expression of cell differentiation markers,^56^ which could be attributed to the release of calcium.^57^ When comparing the ALP activity results from the TiO_2_ + *μ*HA and TiO_2_ + *n*HA coatings, it can be seen that the use of HA nanopowder significantly increases the ALP activity of cells. This could be a combination of two factors: the porous coating morphology and HA particles being more readily dissolved from the coating when the particle size is smaller, thereby providing more sites of local supersaturation with Ca near the surface. The inference about the importance of porous morphology is in good agreement with the results of Yang *et al.* ^36^ showing higher mineralisation activity of osteoblasts cultured for 21 days on a porous HA–TiO_2_ coating compared to both HA-based coating with fused morphology and porous TiO_2_ coatings. As regards to the role of calcium, somewhat conflicting reports about Ca^2+^ ion concentrations required to promote adhesion, proliferation, and differentiation of osteoblast-like cells *in vitro* are noteworthy. For instance, these characteristics were observed to be significantly higher for human fetal osteoblasts-like cell on the surfaces of Ti implants modified with Ca^2+^ ions.^58^ Improved osteoblast adhesion and proliferation on the surface of CaP with higher Ca/P ratios were also reported^59^ and attributed to high Ca^2+^ ion concentration due to the supersaturated condition.^60^ In contrast, different Ca concentrations in the cell culture medium were shown to have no effect on the proliferation of osteoblasts, but higher Ca concentrations enhance the mineralisation.^61^ On the other hand, excessive amounts of Ca^2+^ ions released from dissolving films may result in negative effects on living bone cells.^62^ Even low Ca^2+^ concentrations in the culture medium were shown to induce an increase in ALP activity and osteocalcin mRNA expression in mouse primary osteoblasts.^63^ Our results (Fig. 8) indicate that in the case of the undoped TiO_2_ coating, the calcium phosphate phase has been formed only by calcium adsorption from SBF; therefore, the Ca concentration on the surface was relatively low. For *n*HA-doped coating, the Ca concentration was considerably higher, indicating that additional Ca^2+^ ions released from the coating promoted fast apatite formation, which had a positive effect on osteoblastic differentiation (Fig. 9d). Detailed understanding of the effects of Ca ions on specific aspects of cellular behaviour on such surfaces would require a separate investigation which lies outside the scope of this study.

## 4. Conclusions

This work has investigated the effects of HA particle size in single-stage PEO with the two-step control of electrical parameters on the coating characteristics and biological properties. It has been demonstrated that both micro- and nanoparticles can be successfully incorporated from electrolyte suspensions into the growing coatings although this would result in different surface morphology and distribution of chemical elements across the coating. The micro-particles deposited on the surface altered the porous morphology of the TiO_2_ matrix, reducing the pore size and surface roughness, which led to a highly irregular morphology formed by interpenetrating networks of the voids and the coating material. The nanoparticles migrated more readily towards the surface and penetrated into the coating, partly filling the pores but not altering significantly the porous matrix morphology. Regardless of the particle size, HA increased the coatings' scratch resistance, however cohesive fracture remained the main failure mechanism for all of the studied PEO coatings.

All coatings showed good bioactivity and biocompatibility *in vitro*. The coatings' surfaces were bioactive *in vitro* and induced formation of apatite precipitates during exposure in SBF. The MC3T3-E1 osteoblastic cells were well spread and their actin cytoskeleton was well organised. The cells had a high rate of proliferation on all examined coatings and expressed early-stage differentiation marker alkaline phosphatise. At the same time, the cells' ALP activity after 14 days was significantly higher on the TiO_2_ + *n*HA coating compared to both TiO_2_ and TiO_2_ + *μ*HA coatings. This is likely to be due to the combination of more abundant presence of HA nanoparticles on the surface, as evidenced by corresponding FTIR spectrum rich of specific phosphate bands, and characteristic porous morphology, which provided a synergic effect on the osteoblastic differentiation.
